# Immune checkpoint inhibitor-induced inflammatory arthritis

**DOI:** 10.1016/j.clinme.2025.100496

**Published:** 2025-08-06

**Authors:** Benjamin A. Fisher, Andrew Allard, Shirish Dubey, Kulveer Mankia, Arthur G Pratt, Lalit Pallan

**Affiliations:** a Rheumatology Research Group, Department of Inflammation and Ageing, School of Infection, Inflammation and Immunology, University of Birmingham, Birmingham, UK; b Department of Rheumatology and NIHR Birmingham Biomedical Research Centre, University Hospitals Birmingham NHS Foundation Trust, Birmingham, UK; c Royal National Hospital for Rheumatic Diseases, Royal United Hospitals Bath NHSFT, Bath BA1 3NG, UK; d Dept of Rheumatology, Oxford University Hospitals NHS FT, Oxford OX3 7HE, UK; e Nuffield Department of Orthopaedics, Rheumatology and Musculoskeletal Sciences, University of Oxford, Oxford OX3 7LD, UK; f Leeds Institute of Rheumatic and Musculoskeletal Medicine, University of Leeds, Leeds UK; g NIHR Leeds Biomedical Research Centre, Leeds Teaching Hospitals NHS Trust, Chapel Allerton Hospital, Leeds, UK; h Translational and Clinical Research Institute, Newcastle University, Newcastle upon Tyne, UK; i NIHR Newcastle Biomedical Research Centre, Newcastle upon Tyne Hospitals and Newcastle University, Newcastle upon Tyne, UK; j Department of Oncology, University Hospitals NHS Foundation Trust, Birmingham, UK

**Keywords:** Immune checkpoint inhibitors, Cancer, Arthritis, Adverse events, Anti-tnf

## Abstract

Immune checkpoint inhibitors (ICI) used for the treatment of malignancy are associated with immune-related adverse events, which include inflammatory arthritis. ICI-induced inflammatory arthritis (ICI-IA) is a new clinical entity that may lead to functional impairment and may be persistent even after ICI cessation. We discuss the clinical features, investigation and differential diagnosis. Management needs to consider the safety of immunosuppression in the context of the underlying cancer, and current practice will be further informed by ongoing clinical trials.

## Highlights

• New onset inflammatory arthritis may be triggered by treatment of cancer with immune checkpoint inhibitors (ICI).
• In many cases inflammatory arthritis persists, even after stopping the ICI.
• Multidisciplinary care with referral or joint working with rheumatology is recommended.
• Guidelines typically recommend initial management with glucocorticoids, although dose should ideally be minimised.
• Future guidelines will be informed by the results of ongoing clinical trials.

## Introduction

Immune-checkpoint inhibitors (ICI) (Table 1) have been transformative in cancer care, in some cases allowing long-term survival in previously incurable disease. By blockading inhibitory receptors on T cells, they increase the chances of cancer cell clearance by the immune system. However, ICI are also associated with off-target rheumatological immune-related adverse events (IrAEs) such as arthralgia, myalgia, inflammatory arthritis, myositis, polymyalgia type syndrome, vasculitis, sicca syndrome, scleroderma and rapid bone loss resulting in fractures.^1^^,^^2^ ICI-induced inflammatory arthritis (ICI-IA) occurs in 5–7% of treated patients,^3^ although joint pain in the absence of synovitis (arthralgia) is reported in >40%.^4^ Due to growing ICI use, ICI-IA is an increasingly frequent problem.

**Table 1:** Currently licenced immune checkpoint inhibitors.

Target	Name
CTLA-4	Ipilimumab Tremelimumab
PD-1	Pembrolizumab Nivolumab
PD-L1	Atezolizumab Durvalumab Avelumab
LAG-3	Relatlimab

ICI-IA results in functional impairment, and is often persistent, even after ICI discontinuation.^5^ Studies suggest that at 12 months following discontinuation of immunotherapy, around 50% of patients will still have ongoing inflammatory arthritis.^6^ ICI-IA has a large emotional and quality of life impact.^7^ The uncertainty around arthritis management and the unknown impact of ongoing immunosuppression or ICI cessation on cancer outcomes, further heightens patient anxiety. Thus, ICI-IA is an undesirable outcome during cancer treatment and for cancer survivors.

## Clinical characteristics and differential diagnosis

Inflammatory arthritis is characterised by pain and swelling of synovial joints, morning stiffness that typically lasts an hour or longer, tenderness and loss of function. Unlike rheumatoid arthritis (RA), ICI-IA is not female predominant, with a slight bias towards males.^8^ It is not associated with known RA autoantibodies,^9^ although low- to moderate-titre anti-nuclear antibodies (ANA) may occur in up to a third of cases.^8^ Risk factors for ICI-IA appear to include underlying melanoma, genitourinary tract cancer and pre-existing non-rheumatic autoimmune disease,^10^ which is not an absolute contraindication to ICI use.

Time to onset of arthritis following ICI initiation is variable and relatively late compared to some IrAEs, with a median of 5 months and cases even being observed following ICI cessation.^11^ Around half of patients present with a polyarthritis that includes the small joints of the hands. However, it may also present as an asymmetric oligoarthritis (up to four swollen joints), typically involving medium to large joints such as the knees, ankles and elbows, or a monoarthritis often of the knees. Intriguingly, patients with oligoarthritis sometimes have involvement of joints where there has been a previous injury.

The differential diagnosis will include the more common forms of inflammatory arthritis such as RA. When presenting as a sudden-onset monoarthritis, important differentials will include crystal-mediated and septic arthritis. Reid *et al* have described a phenomenon that they termed ‘activated’ osteoarthritis (ICI-aOA),^12^ affecting joints typically involved by OA with symptoms worse on activity, improved by rest and without significant morning stiffness or joint swelling. ICI can also induce a PMR-like syndrome with hip and particularly shoulder girdle stiffness that may be associated with minor joint swelling or bursal involvement. Remitting seronegative symmetrical synovitis with pitting oedema (RS3PE) can occasionally be observed following ICI and is characterised by puffy swelling and pitting oedema of the hands and often feet, and which will usually respond to steroid treatment alone. The symptoms and signs of inflammatory arthritis should be carefully distinguished from those of neuropathy or myositis that may also occur following ICI. ICI may also induce sarcoid and rarely eosinophilic fasciitis characterised by peau d’orange skin. Given the population being treated, physicians need to be alert to the possibility of metastasis.

## Investigations

**Fig. 1:**
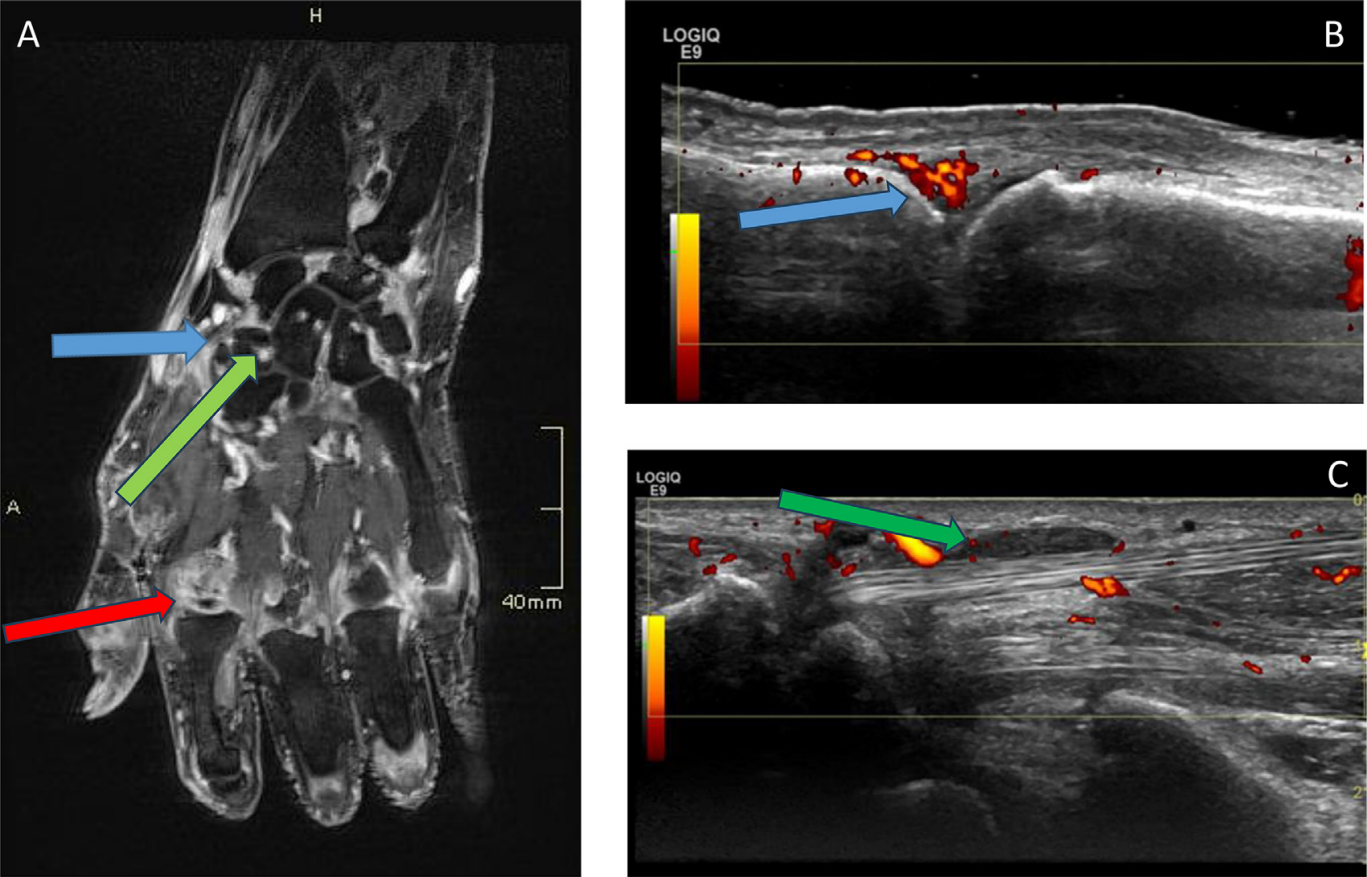
Examples of imaging in patients with ICI-IA. A, 3T MRI scan of hand and wrist using gadolinium contrast. Arrows show extensive synovitis at the wrist and metacarpophalangeal joints with erosions (blue, red and green arrows respectively). B, ultrasound of metacarpophalangeal joint with grade 3 power Doppler signal and isoechoic synovitis (blue arrow). C, Ultrasound showing tenosynovitis of flexor carpi radialis (green arrow). Images courtesy of Dr Mankia.

Investigations are done to distinguish ICI-IA from other de novo rheumatic diseases such as gout, RA, systemic lupus erythematosus (SLE) / connective tissue disease (CTD) and myositis. Blood tests may include CRP, urate, rheumatoid factor, anti-cyclic citrullinated peptide (CCP) antibodies (associated with RA), ANA and creatine kinase (CK). Troponin can be added if clinical suspicion of myositis or myocarditis, which frequently co-occur with one another. A clinical diagnosis of inflammatory synovitis is sometimes obvious, and imaging may not be required. However, with involvement of small joints, X-rays of the hands and feet may establish whether there are pre-existing erosions or osteoarthritic change. Ultrasound may confirm synovitis and/or tenosynovitis (Fig. 1).^13^ MRI may show synovitis, tenosynovitis, enthesitis, effusions and bone marrow oedema and in some cases a predominant pattern of periarticular changes and myofasciitis.^14^ There is a high prevalence of subclinical inflammation on imaging, suggesting that many patients who develop arthralgia after exposure to ICIs may have underlying joint inflammation.^15^ Recent oncology imaging such as PET-CT may also be helpful in providing supportive evidence of inflammatory activity in the joints. Other than for research, synovial fluid examination may add little unless there is a need to exclude crystal-mediated disease or septic arthritis.

## Treatment

Given the potential impact upon quality of life and lack of spontaneous resolution in many cases, treatment is warranted for all but the mildest cases. The goal of treatment is to suppress joint inflammation and, where possible, facilitate ongoing anti-cancer treatment. Guidelines have been published by various organisations,^16^, ^17^, ^18^ although these are based on eminence rather than high-quality evidence. Recommendations from these guidelines are summarised in Table 2 according to severity grade. However, this grading may not optimally capture the nuances of rheumatological assessment and, in general, referral to a rheumatologist is recommended for any patient with persistent joint swelling and/or severe arthralgia.^19^ Tertiary centres often have rheumatologists with expertise or special interest in ICI-IA. Treatment decisions should be made in close collaboration with the oncologists.

**Table 2:** Summary of guidelines for the management of ICI-IA.

CTCAE grade	Description	ASCO	ESMO	SITC
1	Mild pain with inflammation, erythema, or joint swelling	Analgesia with paracetamol and/or NSAIDs	Analgesics and/or NSAIDs	
2	Moderate pain associated with signs of inflammation, erythema, or joint swelling, limiting instrumental ADL	Consider holding ICI. Escalate analgesia If inadequately controlled, prednisone 10–20 mg/day. If no improvement after initial 4 weeks treat as G3. If unable to lower corticosteroid dose to below 10 mg/day after 6–8 weeks, consider DMARD. Consider intra-articular steroid injections for large joints.	Prednisone 10–20 mg/day and taper. Consider higher dose if no response Consider intra-articular injections if mono- or oligo- arthritis No response, consider conventional DMARD, and then biological DMARD if no response	Withold ICI Prednisone 10–20 mg/day Requirement for long-term treatment or failure to respond: conventional or biological DMARDs
3	Severe pain associated with signs of inflammation, erythema, or joint swelling; irreversible joint damage; disabling; limiting self-care ADL	Hold ICI. Initiate oral prednisone 0.5–1 mg/kg. If failure of improvement after 2 weeks or worsening in meantime, consider synthetic or biologic DMARD.	As for grade 2, but withhold ICI	Prednisone 40–60 mg/day Requirement for long-term treatment or failure to respond: conventional or biological DMARDs

ADL, activities of daily living; ASCO, American Society of Clinical Oncology; CTCAE, Common Terminology Criteria for Adverse events; DMARD, disease-modifying anti-rheumatic drug; ESMO, European Society of Medical Oncology; ICI, immune checkpoint inhibitor; SITC, Society for Immunotherapy of Cancer.

Across all guidelines, first-line treatment for patients with swollen joints is glucocorticoids. If one or two joints only are affected, then a trial of intra-articular steroid injection may be reasonable, with oral therapy reserved for failure to respond or a rapid relapse. The starting dose of oral prednisolone varies widely, but may be 10–40 mg depending on severity. This should be titrated down over a few weeks and if this fails to control the arthritis, or there is inability to reduce below 10 mg prednisolone, then initiation of a conventional synthetic disease modifying anti-rheumatic drug (csDMARD) is recommended. Some rheumatologists may prefer an initial intramuscular dose of glucocorticoids (such as triamcinolone) in mild polyarticular disease and/or to initiate csDMARD earlier with very active disease. A typical first csDMARD choice would be weekly oral methotrexate, with a less-used alternative being leflunomide. In mild cases, hydroxychloroquine may be used and this may also be combined with methotrexate. All the cited guidelines mention sulfasalazine as an option. However, sulfasalazine has been associated with T-cell-mediated drug hypersensitivity, known risk factors for which include drug dose and T-cell activation thresholds. We found a high frequency of drug hypersensitivity with sulfasalazine when used in ICI-IA^20^ and supported by *in vitro* data.^21^ Failure of csDMARDs may warrant introduction of biological DMARDs and most experience is with TNF or IL-6R inhibitors. These can be highly effective in controlling symptoms and signs. Guidelines recommend considering withholding ICI with grade 2 moderate severity ICI-IA, in liaison with the treating oncologist.

## Safety of immunosuppression

The objective of ICI treatment is to enhance immune-mediated cancer clearance, hence the possibility that immunosuppression may inhibit this anti-cancer effect needs to be discussed carefully with patients. Many studies have suggested those with IrAEs do better overall in terms of their cancer outcomes than those without,^6^ although that doesn’t answer the question of whether immunosuppression impacts on cancer outcomes. However, patients with non-small cell lung cancer receiving prednisolone ≥10 mg when they start PD-(L)1 blockade have poorer outcomes,^22^ and recent data indicate that high peak doses of glucocorticoids (but not always cumulative doses) are associated with poorer survival.^23^^,^^24^ These data suggest that minimising glucocorticoid use is ideal. Insufficient data exist on csDMARDs such as methotrexate, but one retrospective study in ICI-IA found that use of TNF or IL-6R inhibitors was associated with faster time to arthritis improvement, but may be associated with shorter time to cancer progression.^25^ However, the number of progressing patients in this study was small and the analysis was not corrected for arthritis severity or for peak steroid dose, which was higher in the bDMARD groups. Conversely, animal model data suggest that TNF may be a cancer immune escape pathway in the context of an inflamed tumour microenvironment and confer resistance to anti-PD-1 therapy. In these models, TNF inhibition improves both cancer survival and immune-related toxicity.^26^ A similar hypothesis exists for IL-6 inhibition and small trials published as abstracts with certolizumb (TNFi; NCT03293784) or tocilizumab (IL-6Ri; NCT03999749) given in combination with ICI at initiation have suggested good safety and potential for efficacy. We would advise caution in the use of Janus kinase inhibitors (JAKi) given their possible association with increased risk of cancer^27^ and the important role of interferon gamma in ICI responses. However, recent small trials combining ICI with a short course of JAKi at initiation have suggested better cancer outcomes,^28^ highlighting the complexity of decision-making and need for more data. Abatacept use has generally been avoided in ICI-IA as it inhibits the T-cell co-stimulation pathway targeted by anti-CTLA-4 drugs, so theoretically reducing the effectiveness of ICI.

Ultimately, high-quality randomised evidence is required to guide management of ICI-IA. Some trials have now initiated including the NIHR EME-funded REACT clinical trial in the UK (REmission induction of Arthritis caused by Cancer ImmunoTherapy; ISRCTN18217497). REACT compares standard of care beginning with initial glucocorticoids to the TNFi adalimumab given without glucocorticoids, for the initial systemic treatment of ICI-IA. In RA there is a concept of a ‘window of opportunity’ in early disease, in which effective treatment may modify later disease course. It is unknown yet whether early effective targeted treatment in ICI-IA may increase the eventual rates of drug-free arthritis remission.

## CRediT authorship contribution statement

**Benjamin A. Fisher:** Conceptualization, Writing – original draft. **Andrew Allard:** Writing – review & editing. **Shirish Dubey:** Writing – review & editing. **Kulveer Mankia:** Writing – review & editing, Visualization. **Arthur G Pratt:** Writing – review & editing. **Lalit Pallan:** Writing – review & editing.

## Declaration of competing interests

The authors declare the following financial interests/personal relationships which may be considered as potential competing interests: Lalit Pallan reports a relationship with Bristol Myers Squibb Co that includes: consulting or advisory and speaking and lecture fees. Lalit Pallan reports a relationship with Pierre Fabre SA that includes: travel reimbursement. BAF has received support from the National Institute for Health and Care Research (NIHR) Birmingham Biomedical Research Centre and is chief investigator for the REACT clinical trial. The views expressed in this publication are those of the authors and not necessarily those of the NHS, the NIHR or the Department of Health. BAF has undertaken consultancy for Novartis, BMS, Servier, Galapagos, Roche, UCB, Sanofi, Janssen, AstraZeneca, Otsuka, Amgen, Kiniksa, Cullinan; received research funding from Janssen, Servier, Galapagos, Celgene, Novartis; and speakers fees from Novartis, Servier, Otsuka; all unrelated to the content of the article. AA has received speakers fees from Abbvie, BMS, Eli Lilly, Novartis, Pfizer, Roche, UCB, unrelated to the content of the article. SD has undertaken advisory boards for Abbvie and Boehringer Ingelheim, unrelated to the content of this article. AP and KM have nothing to declare If there are other authors, they declare that they have no known competing financial interests or personal relationships that could have appeared to influence the work reported in this paper.
